# A model for the *Escherichia coli *FtsB/FtsL/FtsQ cell division complex

**DOI:** 10.1186/1472-6807-11-28

**Published:** 2011-06-14

**Authors:** Felipe Villanelo, Alexis Ordenes, Juan Brunet, Rosalba Lagos, Octavio Monasterio

**Affiliations:** 1 Laboratorio de Biología Estructural y Molecular, Departamento de Biología, Facultad de Ciencias, Universidad de Chile. Chile; 2 Instituto de Química, Facultad de Ciencias, Pontificia Universidad Católica de Valparaíso. Chile

## Abstract

**Background:**

Bacterial division is produced by the formation of a macromolecular complex in the middle of the cell, called the *divisome*, formed by more than 10 proteins. This process can be divided into two steps, in which the first is the polymerization of FtsZ to form the Z ring in the cytoplasm, and then the sequential addition of FtsA/ZipA to anchor the ring at the cytoplasmic membrane, a stage completed by FtsEX and FtsK. In the second step, the formation of the peptidoglycan synthesis machinery in the periplasm takes place, followed by cell division. The proteins involved in connecting both steps in cell division are FtsQ, FtsB and FtsL, and their interaction is a crucial and conserved event in the division of different bacteria. These components are small bitopic membrane proteins, and their specific function seems to be mainly structural. The purpose of this study was to obtain a structural model of the periplasmic part of the FtsB/FtsL/FtsQ complex, using bioinformatics tools and experimental data reported in the literature.

**Results:**

Two oligomeric models for the periplasmic region of the FtsB/FtsL/FtsQ *E. coli *complex were obtained from bioinformatics analysis. The FtsB/FtsL subcomplex was modelled as a coiled-coil based on sequence information and several stoichiometric possibilities. The crystallographic structure of FtsQ was added to this complex, through protein-protein docking. Two final structurally-stable models, one trimeric and one hexameric, were obtained. The nature of the protein-protein contacts was energetically favourable in both models and the overall structures were in agreement with the experimental evidence reported.

**Conclusions:**

The two models obtained for the FtsB/FtsL/FtsQ complex were stable and thus compatible with the *in vivo *periplasmic complex structure. Although the hexameric model 2:2:2 has features that indicate that this is the most plausible structure, the ternary complex 1:1:1 cannot be discarded. Both models could be further stabilized by the binding of the other proteins of the *divisome*. The bioinformatics modelling of this kind of protein complex, whose function is mainly structural, provide useful information. Experimental results should confirm or reject these models and provide new data for future bioinformatics studies to refine the models.

## Background

Bacterial cell division is performed at the middle of the cell, after duplication and segregation of the genetic material into the daughter nucleoids. In *Escherichia coli*, this process requires at least 12 essential proteins, localized at the constriction site at the cell equator. These proteins coordinate the invagination of the cytoplasmic membrane and guide the inward growth of the peptidoglycan to produce the daughter cells. The proteins FtsZ, FtsA, ZipA, FtsE/FtsX, FtsK, FtsQ, FtsB/FtsL, FtsW, FtsI and FtsN have been identified mainly through microscopy observation of GFP-protein fusions and deletions of the corresponding gene (reviewed in [1] and [2]).

The *E. coli divisome*, the macromolecular complex composed of the aforementioned proteins, is assembled in an almost sequential way. FtsZ polymerization is the leading event, recruiting proteins such as FtsA and ZipA that attach the polymer to the inner face of the cytoplasmic membrane. The proteins are recruited in the following order: FtsZ > FtsA/ZipA > FtsE/FtsX > FtsK > FtsQ > FtsB/FtsL > FtsW > FtsI > FtsN [3-5]. The recruiting mechanism, the binding characteristics, and the exact function of some of these proteins is still unknown.

**Figure 1:**
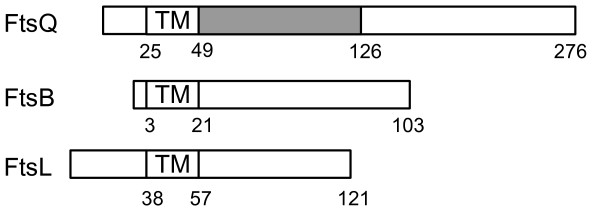
**Topology of FtsB, FtsL and FtsQ**. Schematic topology of FtsB, FtsL and FtsQ, with their transmembrane regions (TM) aligned. The POTRA-like domain of FtsQ is greyed. The scheme is not drawn to scale.

FtsQ is a low abundance periplasmic protein in *E. coli *(~22 copies per cell) [6], composed of 276 residues with a bitopic membrane topology (Figure 1). The structure includes a short cytoplasmic N-terminal tail, a membrane-spanning helix, and a longer 226-residue periplasmic section indispensable for the division process [7,8]. This protein seems to have a central role in divisome formation, but its exact functional properties remain unknown. FtsQ localization in the divisome depends on FtsK [9], and drives the localization of the subsequent proteins, including FtsB, FtsL, FtsI, FtsW and FtsN [10-14]. The periplasmic part of FtsQ consists of two domains called alpha and beta [PDB:2VH1] as described in the crystal structure of the *E. coli *and *Yersinia enterocolitica *proteins [15]. The alpha domain corresponds to a POTRA domain presumably involved in chaperone-like functions that was first predicted from sequence analysis [16]. The beta domain, includes a region involved in the interactions with FtsB/FtsL, whose structure was determined by NMR for the *Geobacillus stearothermophilus *FtsQ-homologue DivIB [17]. The last 30-40 C-terminal residues are non-structured and they seem to be determinant for the interaction with FtsB/FtsL in *E. coli *[18,19]. This sequence was proposed as a domain from limited proteolysis analysis [17,20]. However this is not a separated domain, as shown by the crystal structure, because these residues are part of the long, extented beta sheet comprising the beta domain.

The alpha domain, immediately after the transmembrane helix, is very similar to a POTRA domain of other reported structures, confirming the predictions of Sanchez-Pulido et al. [16]. This domain could protect FtsB/FtsL from denaturation or degradation although a chaperone-activity has not been probed. Site directed mutations of FtsQ (V92D, Q108L, V111G, K113D, E125K) indicate that this domain is the FtsK-interacting part of the protein, allowing its correct localization in the *divisome *[15,19,21]. The second domain is structured as a beta sheet surrounded by two twisted helices. Mutations in this domain (Q232R, D237N, A252P and L259S) prevent the recruitment of FtsB/FtsL [15,19,22]. The third non-structured domain proposed for the *G. stearothermophilus *FtsQ homologue DivIB [17] is part of the second domain in the crystallographic *E. coli *and *Y. enterocolitica *structures, rather than as an independent domain [15].

FtsB and FtsL are short proteins (121 and 103 residues respectively) with a topology similar to FtsQ (Figure 1). Both proteins have a leucine heptad in their periplasmic region, suggesting the presence of a leucine zipper motif, typical of coiled-coil proteins [10,23]. The homologues of these proteins in Gram-positive bacteria, FtsL and DivIC respectively, share these characteristics [24,25]. FtsL associates with itself forming unstable dimers *in vitro *and as heterocomplexes with FtsB and FtsQ at the division site or elsewhere [10,23,26]. The recruitment of FtsB and FtsL to the *E. coli divisome *depends on their mutual interaction [26]. These specific interactions have been mapped using protein truncation experiments, highlighting the importance of the C-terminal regions of FtsB and FtsL for the interaction with FtsQ, and the need of the FtsL cytoplasmic tail for the interaction with FtsW [27,28]. In *Bacillus subtilis *DivIC (FtsB) and FtsL are unstable proteins which are stabilized when in contact with each other and with DivIB at the septum (FtsQ homologue) [29,30]. It has been proposed that *B. subtilis *FtsL instability could be a control point in divisome formation, and that the membrane metalloprotease YluC is involved in the degradation of the complex [31]. This membrane protease, which participates in the regulated intramembrane proteolysis (RIP) process, has a homologue in *E. coli *called RseP, which can hydrolyze unstable membrane-spanning domains in some proteins [32,33].

The FtsB/FtsL/FtsQ heterocomplex in *E. coli *exists as a late recruitment event, together with proteins involved in cell wall synthesis [3,26]. Corresponding genes have been identified in the genomes of many different bacteria [28]. This complex was isolated in *E. coli *by co-immunoprecipitation [26] and the interaction has been confirmed by other methods [4,5,34]. The structure of this complex is a subject of interest in many laboratories, because it seems to have a crucial structural function in cell division. Masson et al. [18] proposed a low resolution structure of this ternary complex based on analysis by NMR, surface plasmon resonance, small-angle neutron and X-ray scattering using the protein homologues from *Streptococcus pneumoniae *(FtsL, DivIC and DivIB). In this study, the POTRA domain of FtsQ appears loosely-structured and the main interactions between these proteins are through the C-terminal region, leaving the coiled-coil part of DivIC(FtsB)/FtsL free to interact with other proteins. The extracellular regions of DivIC(FtsB) and FtsL do not interact *in vitro *so this interaction was forced by fusing each protein to a coiled-coil peptide, leading to the formation of a putative heterodimer complex with a 1:1 DivIC/FtsL ratio [18,34]. The three-protein complex could then be formed independent of FtsK [26] and its formation is probably crucial in order to stabilize FtsL and FtsB through the interaction with FtsQ, like in *B. subtilis *[29,35]. The binding of FtsQ to the FtsB/FtsL complex could also be important for its stoichiometry [1,15].

In this work, we propose the most probable atomic structures of the ternary FtsB/FtsL/FtsQ complex, its stoichiometry and the nature of their interactions. The most probable and stable models were the trimeric and hexameric complexes, with proteins in a 1:1:1 ratio. These models could help to understand a crucial step in *divisome *formation, the importance of this complex in the sequential binding of the other proteins and their role in the control of the division process. The theoretical construction of this complex could be useful to design experiments in order to confirm these models and to make progress in the understanding of the bacterial division process.

## Results and discussion

### FtsB and FtsL monomer modelling

**Figure 2:**
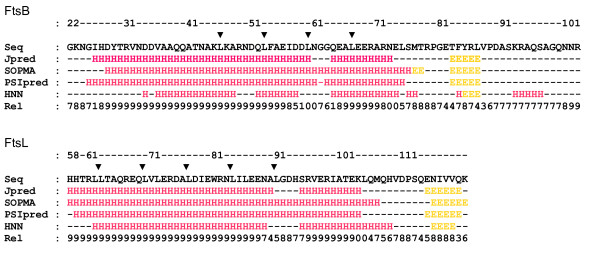
**Secondary structure prediction of the periplasmic region of FtsB and FtsL**. The prediction was made with the Jpred3, SOMPA, PSIpred and HNN servers. The leucine residues involved in the zipper motif are indicated with arrowhead. Seq: primary sequence; Server name: secondary structure prediction (H: helix; E: extended; -: neither helix nor extended); Rel: reliability of the prediction averaged between the normalized reliability reported by each server used.

These division proteins were previously reported as possible coiled-coil proteins, as predicted by the COILS program and the presence of a leucine heptad fragment in the periplasmic region, which could form a leucine zipper motif [10,23]. Secondary structure predictions show that both proteins have a long helix between the membrane-spanning region and the C-terminal region, which could include a loop (Figure 2). The sequence of both proteins is not well conserved in other bacteria, but the leucine zipper motif and other structural characteristics are conserved [28]. It is important to mention the conservation of the leucine residues and the high proportion of charged residues (Additional File 1: Figures S1 and S2).

In FtsL, the leucine zipper motif extends from close to the membrane-spanning helix to approximately residue 92 [28]. Here two of four secondary structure predictions show a break in the helix (Figure 2), where the residues GDHS could form a turn, followed by a second helix (residues 96 to 105). This observation is not conclusive because the residues in the proposed helix-breaking turn are not fully conserved (Additional File 1: Figure S2). Besides, one of the other two secondary structure prediction (SOMPA) shown in Figure 2, has a high reliability for the helix prediction in these residues. After these residues, neither leucine nor other hydrophobic residues are observed in the correct position in the helix to form the coiled-coil interaction. The presence of this possible turn is important because in the hexameric FtsB/FtsL/FtsQ model, a helix-break is indeed modelled in FtsL in order to fit correctly with the other structures. However, for the other models this is not necessary because a straight helix is sufficient to model FtsL. In FtsB, leucine residues are present in the distal region with respect to the membrane-spanning helix, from position 46 to 75. In this protein, the secondary structure prediction shows a helix configuration from the transmembrane helix to residue 78. For both proteins, the helix prediction includes the leucine zipper motif, but in the case of FtsB, it is much longer (Figure 2).

The last 12 residues of FtsL (109-121) were not modelled due to the null helical tendency and therefore the lack of an adequate template structure in the PDB database for homology modelling. These C-terminal residues in the model include residues defined as necessary for the FtsQ interaction [28]. In the last 20 amino acids of FtsB (83-103), the secondary structure prediction is similar to FtsL, the helix tendency is lost and there is no adequate template in the PDB database, although a strand structure is predicted between residues 83 and 87. This part of the FtsB model includes the residues reported to interact with FtsQ [27]. The crystallographic structures selected for both proteins as template came from threading searches using the secondary structure described before (details in Methods section).

### Modelling different stoichiometries

There is a lack of experimental data regarding the stoichiometry of the FtsB/FtsL/FtsQ complex. FtsB/FtsL proteins form a complex prior to the binding of FtsQ through coiled-coil interactions [10,23,26], and for this reason the FtsB/FtsL complex was modelled first, and then FtsQ was added. Different oligomeric possibilities observed in coiled-coil multimers were assayed for the FtsB/FtsL complex e.g. dimer, trimer and tetramer. The pentamer was not considered due to the lack of bulky hydrophobic residues, such as tryptophan or tyrosine necessary to stabilize this type of protein-protein interaction [36,37]. In FtsB or FtsL, there are few such residues and they are not in an appropriate position to make a possible interacting interface between helices.

The strategy used for the stoichiometry analysis was to model several oligomeric complexes and then to determine their structural stability by molecular dynamics simulations. The leucine heptad is crucial in order to form the zipper motif during the modelling of the complex; notwithstanding, there are other important residues in this type of binding and in the stoichiometry of the multimer [38]. FtsB has the leucine residues in its distal periplasmic region, and FtsL in its proximal region with respect to the lipid bilayer, but this coiled-coil motif could expand along the helix because the leucines that form the zipper motif can be replaced by other hydrophobic residues such as isoleucine or valine. This fact is important for modelling complexes with different stoichiometries, but sequence analysis was not considered for the stoichiometry, because of the random distribution of important interaction residues reported for coiled-coil interactions, except for the leucines. In several studies, a pattern of residues has been identified in particular stoichiometries of coiled-coil folds [39-42] but multiple alignments of several FtsB and FtsL sequences did not show specific amino acids in the right position of the sequence to give a known stoichiometry pattern (Additional File 1: Figures S1 and S2).

**Figure 3:**
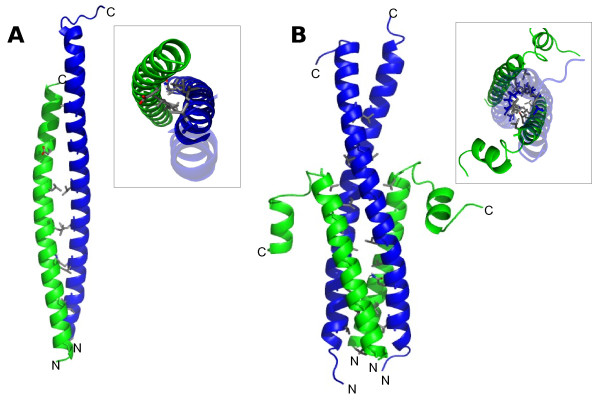
**FtsB/FtsL complex models**. Modelled structures of the FtsB/FtsL complex before the addition of FtsQ. In blue FtsB, in green FtsL. **A**. Heterodimer FtsB/FtsL; **B**. Heterotetramer 2FtsB/2FtsL. The insets are top views (from C-terminal to N-terminal) of each model. The residues of the position *a *in the coiled-coil are shown as sticks, coloured by atom type (carbon in grey, oxygen in red, nitrogen in blue). The majority of these residues are leucines (see the text for explanation). The range of residues modelled is 25 to 88 for FtsB, and 61 to 109 for FtsL. N- and C-terminals are indicated.

**Figure 4:**
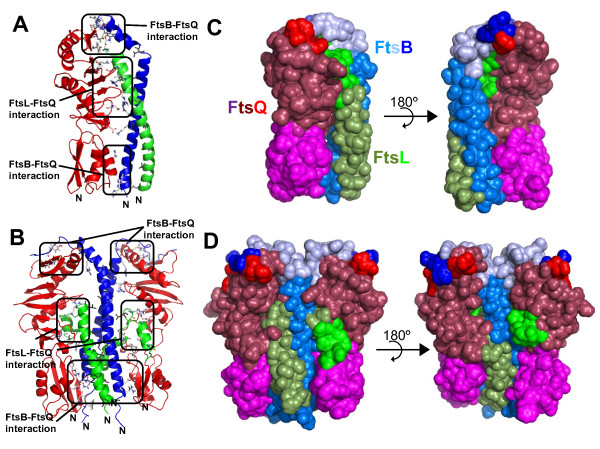
**FtsB/FtsL/FtsQ complex models**. Representative structures of FtsB/FtsL/FtsQ complex models produced during dynamics simulation. **A**. Trimeric 1:1:1 model. FtsB are shown in blue, FtsL in green and FtsQ in red. The interacting residues described in the text are in stick form, coloured by atom type and grouped by the pair of proteins involved in the interaction. Leucines in the zipper between FtsB and FtsL are shown in grey sticks. N indicates the N-terminal residue of each protein. The C-terminals are omitted for clarity, but all three are placed at the top of the figure. **B**. Hexameric 2:2:2 model. The colour coding is the same as explained in A. **C**. Solvent accessible surface of the trimeric model, showing the regions identified previously in the proteins as crucial for the interaction. In purple, the FtsQ POTRA domain (residues 58 to 126); in burgundy, the FtsQ C-terminal domain (residues 127 to 145); in red, the FtsQ residues reported to be essential for FtsB/FtsL recruitment (245 to 260); in pale green, the FtsL coiled-coil region (residues 61 to 95); in green, the FtsL residues reported to be crucial for interaction with FtsQ (96 to 109); in marine blue, the FtsB coiled-coil region (region 25 to 67); in light blue, the FtsB region not identified to participate in any interaction (residues 68 to 84); in blue, the FtsB region identified to interact with FtsQ (residues 84 to 88); in grey, the leucine residues involved in the leucine-zipper between FtsB and FtsL. **D**. Solvent accessible surface of the hexameric model, with the same colour coding as above. In C and D, the left panels correspond to the same view as A and B, and the right panels correspond to a 180° rotated view.

Figure 3A shows the dimeric FtsB/FtsL complex as a large coiled-coil structure of 78 Å, with interactions between both proteins along the helices. The middle section of this complex is stabilized by the leucine zipper and in the distal and proximal sections, the interacting residues are glutamines, valines and alanines, in the positions *a *and *d *of the coiled-coil where there are no leucine residues. The last C-terminal residues of FtsB remain free, as the FtsL periplasmic domain is shorter. Molecular dynamics simulation of this binary complex shows instability and high flexibility, but the structure roughly maintains its coiled-coil configuration. It is important to mention that the FtsL monomer was taken as a long helix without the helix-break turn in residue 92, because if this turn was considered, the C-terminal region after the break would remain completely solvent-exposed, without any stabilizing interactions (data not shown). For the construction of a three-helix complex of FtsB/FtsL there were two possible combinations: 1FtsB:2FtsL and 2FtsB:1FtsL. In both cases, the construction was similar to the 1:1 model, with all proteins forming long helices. For the four-helix 2FtsB:2FtsL model (Figure 3B), the interaction of a FtsB dimer with a FtsL dimer to form the tetrameric complex was not appropriate and instead two FtsB/FtsL heterodimers were constructed giving rise to a tetramer with more stable interactions in which the leucine residues in the four chains interacted correctly. Interestingly, keeping a straight helix in FtsL monomer lead to steric impediment during the subsequent docking of FtsQ, especially in the C-terminal region, which was the main part for the interaction with FtsQ. Hence, a different conformation of FtsL was used to model the complex, including a turn in the residues GDHS as found in the secondary structure prediction. This new FtsL model allowed the construction of the hexameric 2FtsB:2FtsL:2FtsQ model without steric clashes (Figure 4B).

To model the ternary FtsB/FtsL/FtsQ complex, the different FtsB/FtsL models described above (Figure 3) were used to perform docking experiments with the crystallographic structure of FtsQ. This gave rise to four FtsB/FtsL/FtsQ models with different stoichiometries. In the models with two and three helices in coiled-coils, only one FtsQ molecule was added due to steric impediment, but in the four helix coiled-coils, two FtsQ molecules were added to stabilize the FtsL C-terminal helix. Then, four FtsB/FtsL/FtsQ models were obtained: one trimeric; two tetrameric and one hexameric. With respect to the number of FtsQ molecules in the models, there is contradictory evidence about its multimeric state *in vivo*. Two-hybrid studies of the periplasmic/extracellular domain of FtsQ show that it is a protein capable of self-interaction [3,4,19], but these observations are indirect and have been refuted by more direct experiments, such as co-immunoprecipitation of *E. coli *FtsQ [5], multiangle laser scattering of *G. stearothermophilus *DivIB [17] and analytical ultracentrifugation of various bacterial FtsQ and DivIB [15]. The presence of the membrane-spanning domain could affect this situation *in vivo *and promote oligomerization. Besides, the very limited amount of FtsQ molecules in *E. coli*, reinforces the proposal that FtsQ is a protein that does not self-interact, but that it does form complexes with other proteins, like FtsB/FtsL.

To date, the experimental data on the FtsB/FtsL interaction came mainly from two- or three-hybrid studies, for Gram-positive as well as for Gram-negative bacterial proteins [4,19,30,35] and were confirmed by co-immunoprecipitation of these proteins from *E. coli *[10,26-28]. The immunoprecipitation experiments do not provide useful information on the binding stoichiometry, in contrast with the studies involving the direct observation of homolog proteins from *S. pneumoniae *[18,34], which strongly suggest the formation of heterodimers of FtsL/DivIC(FtsB) in a 1:1 ratio. These observations could be true for *E. coli *proteins, but the low conservation of sequence between them lead us to consider other possibilities. Besides, in the NMR structure of the periplasmic domain of DivIB (FtsQ), the POTRA domain appears loosely-structured, a feature very unlikely under physiological conditions. The authors attribute this to a marginal conformational stability of the protein in some species, so this domain is not stable in conditions that differ from those *in vivo *[18]. This explanation could be extended to the stoichiometry observed, so the 1:1 FtsL/DivIC(FtsB) heterodimer could be distinct in the *in vivo *complex of *E. coli*. This reasoning lead us to consider different ratios between the studied proteins.

### FtsB/FtsL/FtsQ models

Once the possible FtsB/FtsL stoichiometry has been defined, the FtsQ crystallographic structure can be added through a docking procedure (details in Methods section) using experimental data to restrict the number of models obtained. In spite of the abundant information in the literature about mutants in the *ftsQ *gene, the mutations used were those studied in van den Ent et al. [15] because these are point mutations which generate a known phenotype and are concordant with others reported before [19,22]. These residues are Q232, D237 and L259, all in the C-terminal domain, required for FtsB/FtsL recruitment. The FtsB/FtsL/FtsQ models obtained in this way were long and planar, mainly due to the elongated form of both the FtsQ and the FtsB/FtsL coiled-coil folds, being between 75 and 80 Å long (Figure 4). The models were subjected to a molecular dynamic routine to analyse their stability: energy minimization, simulation annealing, equilibration dynamic, and production dynamic of 10 ns. On one hand, the trimeric and hexameric models maintained their structure and showed sufficient stability through the process. On the other hand, for both tetrameric models, which include three helices in the coiled-coil fold, the conformation was lost at some point of the simulation, and the structure was not maintained during the process (data not shown). In the course of the simulation of these models, the solvent molecules diffused into the protein interface, disassembling the complex almost completely and just some secondary structure elements remained. These observations led us to discard the tetrameric models and some minimal calculations were used to confirm this determination. The work detailed below thus relates to the trimeric and hexameric FtsB/FtsL/FtsQ complexes.

In order to simulate the biological conditions, position restraint (an algorithm for maintaining a group of atoms in a fixed position) was applied for the N-terminal main chain atoms in the first residue of all peptide chains, due to the lack of transmembrane helices in the model. With this approach, these residues were fixed to a virtual membrane zone in all the dynamics. During the simulation annealing (going from 200 to 300K), some structural changes occurred but it was not clear if these changes were due to the artificial forces applied in the described restraint or if they corresponded to a real dynamic process. The FtsB and FtsL helices should rotate and translate freely, however the movements were restricted by the rigidity imposed by artificial forces applied that fix the polypeptide chain at the amino terminus. In the real environment, the entire complex should have translational and rotational movement. In order to simulate this movement, a distance restraint should have been applied between the main chain atoms of N-terminal residues of the different chains, but this was not possible with the software used.

**Figure 5:**
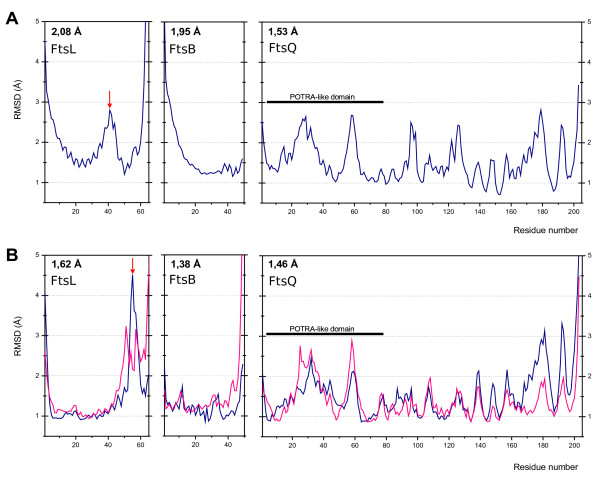
**Flexibility of FtsB/FtsL/FtsQ models**. Root mean square average fluctuation in angstroms of main chain atoms in the FtsB/FtsL/FtsQ models produced during dynamics simulation of 10 ns. The X-axis represents the amino acid sequence position numbering in the models (residue 1 is the first in each model). The average RMS of each protein is detailed in the upper left corner of each box. **A**. Trimeric 1:1:1 model and **B**. Hexameric 2:2:2 model (the blue and pink lines correspond to the two different molecules of the same protein in the complex). The red arrows indicate the maximum mobility of FtsB; in A, a helix break is located at position 66 whereas in B maximum mobility occurs at the end of the coiled-coil helix and prior to the beta-like interaction with FtsQ. See the text for a detailed explanation.

After the 10 ns of molecular dynamics simulation at NpT conditions (number or particles, temperature and pressure are maintained constant), the overall structure of the tertiary FtsB/FtsL/FtsQ complex remained stable in the trimeric and hexameric models with a RMS about 1-2 Å with respect to the initial model (Figure 5). The FtsQ molecules were flexible mainly in the loops that connect the secondary structure elements. FtsB and FtsL retained their structure in the 1-2 Å range with some differences between the two models, especially in FtsB (Figure 5). Although FtsL was not modelled with the same structure in the trimeric and hexameric models, it was very stable in both cases. In both the trimeric and hexameric models, the C-terminal residues of FtsQ and FtsB spontaneously formed a beta-like interaction, although in the hexameric complex, this region was less defined than in the trimeric model. In both cases, these interactions stabilized the exposed residues, lowering their mobility throughout the dynamic process. Hence, the complex FtsB/FtsL binds FtsQ in a specific way. However, in these complexes the C-terminal region was flexible, mainly stabilized by hydrogen bonds that could be disrupted by the solvent due to the polar nature of this interaction. Although conserved aromatic residues such as Phe84 and Tyr85 in FtsB, and Trp256 and Tyr258 in FtsQ could be important in accounting for the binding strength, the simulation showed only *van der Waals *and hydrogen interactions, with no electron dynamics such as aromatic *π-π *interactions that could be important (the simulation, based on classical mechanics, does not consider electron dynamics). In the zone of the complexes proximal to the membrane, the hexameric model showed more interactions than the trimeric complex, where hydrogen bonds and saline bridges maintained the sandwich-like position of FtsQ with respect to the coiled-coil fold. These interactions between FtsB/FtsL coiled-coils and the FtsQ POTRA domain were not reported previously as crucial [18,19,22]. However, they could help to stabilize the complex once the mandatory C-terminal interactions have been formed. In the previous model of the *S. pneumoniae *DivIB(FtsQ)/FtsL/DivIC(FtsB) complex [18], these interactions could have been missed due to the low structured conformation of the POTRA domain at the conditions used in the experiments. Nevertheless, it is important to bear in mind that the stability could be reinforced by the possible interaction of the membrane-spanning helices of these proteins. Thus, the membrane helices of the three proteins could contribute to the interactions, as shown for FtsQ [43]. In our work, the movement restriction imposed by the membrane was simulated through artificial forces (position restraint), but the use of explicit membrane lipid dynamics would be very useful. The dynamic of the lipid bilayer and the residues around the phospholipid headgroups could significantly influence the interactions and stabilities observed in this work and this fact would be relevant to select one of the models.

The elongated forms of both FtsB/FtsL/FtsQ models are in agreement with the model from *S. pneumoniae *DivIB(FtsQ)/FtsL/DivIC(FtsB) [18] where the C-terminal domain (β domain) of DivIB(FtsQ) is the region responsible for the binding of the FtsL/DivIC(FtsB) forced heterodimer (named KL/EC). In the NMR spectrum, the POTRA domain and the γ domain (the last 35 residues) of DivIB(FtsQ) do not change upon FtsL/DivIC(FtsB) binding, but this could be due to the lack of structure of these domains in the assayed conditions. The C-terminal regions of KL/EC interact with the β domain of DivIB(FtsQ), and considering the likely sizes of these proteins let the authors to propose a tilt in the helical coiled-coil interaction. This tilt could exist in the coiled-coil fold itself or at the interface between coiled-coil and transmembrane helices. The latter option is considered to be more likely because the secondary structure prediction of these proteins shows an absence of helical tendency in this region (Figure 2). These conclusions came from the *ab initio *model of the β domain of DivIB(FstQ) interacting with KL/EC, constructed from the SANS (small-angle neutron scattering) distance distribution function. This model includes the KL/EC heterodimer, a forced version of the FtsL/DivIC(FtsB) interaction, which comprises the k5 and e5 peptides and a tag for purification [18]. These fusion versions of the proteins are considerably larger than the extracellular/periplasmic domains of FtsL and DivIC(FtsB), so in the model constructed, the extension of real protein interacting with DivIB(FtsQ) is somewhat difficult to estimate. The contacts between the POTRA domain of FtsQ(DivIB) and FtsL/FtsB(DivIC) observed in our models are missing in the model of Masson et al. [18], and the explanations could be: that the unstructured POTRA domain in the assayed condition impedes the correct interaction with the KL/EC dimer or; that the interaction is of a distinct nature in the two species, one Gram-negative bacteria (*E. coli*), the other Gram-positive bacteria (*S. pneumoniae*). Nevertheless, the overall shape of both models is concordant, and previous experimental observations are consistent with the proposed model.

#### Trimeric model

The trimeric model (Figure 4A) has dimensions of 35, 20 and 79 Å in the x, y and z-axis respectively, where the z-axis is perpendicular to the membrane. The characteristics of the interaction surface between the FtsB/FtsL complex and FtsQ in the trimeric model are reported in Table 1. The FtsQ molecule makes contact with the FtsB/FtsL heterodimer through the beta sheet at the C-terminal domain and helices H1 and H2 in the POTRA domain. The contact of FtsQ with the other proteins occurs via its C-terminal domain and also through the POTRA domain. The contacts identified as "hotspots" are reported (Additional File 2: Table S1). There are several hydrophobic residues of FtsL exposed to the solvent, however the main contact with FtsQ is at the C-terminal region of FtsL (residues 88 to 109) via polar interactions. The specific residues involved in the ionic network at the C-terminal region were: Arg99(FtsL)-Glu190(FtsQ); Glu103(FtsL)-Lys208(FtsQ); Lys45(FtsB)-Glu150(FtsQ) and Arg49(FtsB)-Asp134(FtsQ). There is a little discordance between the model and the experimental data regarding FtsL/FtsQ contacts (Figure 4C), because in the trimeric model, the interacting region of FtsL was displaced towards the N-terminal with respect to the previously-identified region (residues 100 to 114 in FtsL) [28].

**Table 1:** Interface parameters of the complexes

FtsB:FtsL:FtsQ ^**a, b**^	**Interface ASA (Å**^**2**^)	Interface ASA %	**% Polar **^**c**^	**% Non polar **^**c**^	**% Charged **^**c**^	**H-bonds/100 Å**^**2**^	**Salt bridges/100 Å**^**2**^
Trimeric (1:1:1)	1341.32	11.79	30.70	43.60	25.60	0.5	1.90
Tetrameric (1:2:1)	933.29	7.80	23.08	26.92	50.00	0.4	2.68
Tetrameric (2:1:1)	824.55	6.12	26.86	24.53	48.61	0.4	2.11
Hexameric (2:2:2)	4913.82	33.53	26.72	39.66	33.62	0.5	1.34
Mean stable complex ^d^		11.2	32.2	39.5	28.2	1	2 - 6

^a ^The interface parameters were obtained with the PROTORP server for the FtsB/FtsL/FtsQ complex after the molecular dynamic equilibration.

^b ^The interfaces considered were between the FtsB/FtsL subcomplex and FtsQ. The FtsB and FtsL pair was treated as a single unit.

^c ^The percentages of each kind of residue consider only the residues at the interface.

^d ^The data for the mean stable complex were extracted from Reynolds et al. [65]

A beta-sheet-like conformation formed spontaneously between the last C-terminal residues of FtsB (76-88) and the last β-strand of FtsQ (251-258), stabilized by hydrogen bonds. This region in FtsB adopted a twisted beta configuration, establishing several hydrogen bonds with FtsQ. During the rest of the simulation, this beta-like hydrogen interaction remained stable and diminished the flexibility of this zone (Figure 5A). This was an interesting observation because FtsB and FtsQ C-terminal residues were previously-reported as being crucial in this interaction (Figure 4C) [27]. A couple of polar interactions were detected between FtsB and the POTRA domain of FtsQ, such as Glu83(FtsQ)-His27(FtsB) and Gln76(FtsQ)-Asp34(FtsB), whilst the Pro84(FtsQ)-Thr30(FtsB)-Leu70(FtsQ) interaction was mediated by van der Waals forces. These interactions were not reported in Masson et al. [18], as explained above. Another interesting feature found in the dynamic simulation of the FtsB/FtsL/FtsQ complex is a break in the helix of FtsB. This conformational change occurs in residues 65-67, just 5 positions upstream of a break predicted by the secondary structure (Figure 2) that was not considered for the modelling of the FtsB monomer. This break in the helix could be related to the proposed tilt in the coiled-coil fold proposed by Masson et al [18] in order to fit the C-terminal regions.

#### Hexameric model

The dimensions of the hexameric FtsB/FtsL/FtsQ model were 72, 20 and 90 Å for the x, y and z axis respectively, with a symmetrical distribution of protein chains on both sides (Figure 4B). The side of FtsQ facing the FtsB/FtsL heterotetramer is slightly displaced towards helices H4 and H5 in the C-terminal domain, with respect to the trimeric model. The total number of hydrogen bonds found between the FtsB/FtsL complex and FtsQ in the hexameric model were around 27, and the number of salt bridges around 50 (Table 1). This model shows that two molecules of FtsQ were located at opposite sides of the four-helix coiled-coil FtsB/FtsL complex. The most important contacts identified as "hotspots" are reported (Additional File 2: Table S1).

FtsL molecules interact with FtsQ along their longitudinal axis, mainly through the C-terminal helices (residues 94 to 104) that were accommodated between helices H3 and H4 of the C-terminal domain of each FtsQ molecule. This interaction was mediated by hydrogen bonds and salt bridges. The loop between the FtsL helices (residues 89-92), absent in the trimeric model, established several ionic contacts with loops in FtsQ, such as residues 127-158 in the C-terminal domain. One of the FtsL monomers lost the interaction with FtsQ during the dynamic simulation by disruption of the short distal helix during the molecular dynamics simulation. These facts are unexpected due to the reported importance of residues 100-114 of FtsL in the binding of FtsQ [28], but not of the residues in the 89-92 range (Figure 4D). This contradiction between our model and the reported experimental data could be attributable to the unknown conformation of FtsL, that could present a helix-loop-helix conformation (like in the hexameric model) or a straight helix from the membrane-spanning domain to the C-terminal residues (like in the trimeric model). We cannot distinguish between these conformations with the tools used here, and the solving of this divergence would allow us to correctly position the C-terminal residues of FtsL in the model, which could be concordant with the experimental evidence [28].

FtsB molecules interact with FtsQ mainly in two areas: one close to the membrane region, and the other at the C-terminal region. These interactions are the most relevant within the hexameric model. The C-terminal region of FtsB (residues 76 to 88), as explained for the trimeric model, was initially almost unstructured. This situation changed in the equilibration dynamic simulation tending to form a beta-like structure that interacted with the C-terminal beta sheet of FtsQ. However, this beta-like structure was less stable than in the trimeric model during the dynamic simulation, with different behaviour on either side of the hexameric model. On the other hand, the interaction between FtsB and the POTRA domain of FtsQ at the boundaries of the virtual transmembrane region includes salt bridges, hydrogen bonds and *van der Waals *interactions, the last of which could contribute to maintain the stability of the structure. These hydrophobic contacts could indicate an important interaction missed in the model of Masson et al. [20], but not crucial for interaction [27] (Figure 4D). These *van der Waals *contacts were made by Leu60(FtsQ), Tyr68(FtsQ), Val127(FtsQ), Ile129(FtsQ), Ile85(FtsL), Leu86(FtsL), Val97(FtsL), Ile100(FtsL) and Leu105(FtsL). In the middle section of the FtsB molecule (residues 46 to 78), there were only *van der Waals *interactions with their FtsL partners which stabilized the heterotetrameric interface via the amino acid residues leucine 60, 67 and 75 of the zipper motif.

The molecular dynamic simulation showed that during the equilibration process, the central part of one FtsB molecule presents a break in the helical configuration, at position 45, probably caused by the position restraint of the N-terminal residues. However, this helix disruption did not interfere with the stability of the complex. The same occurred with FtsL, specifically in one molecule, resulting in a twisted and disordered helix that interacts in a different way with FtsQ. The break in the helical conformations during the simulations is due to the need to fit the positions between the C-terminal regions of FtsB and FtsQ. The POTRA domain of FtsQ showed more flexibility than in the trimeric model, being different for the two monomers of this molecule (Figure 5B). The flexibility of the POTRA domain could be in agreement with the observations for the *S. pneumoniae *FtsQ homologue DivIB, where this domain appears loosely-structured [20]. Nevertheless, this augmented flexibility is marginal, and the contacts described between FtsQ and FtsB were the most stable part within the model. The region of the hexameric complex located around the POTRA domain in FtsQ showed that the tetrameric coiled-coil structure between FtsB and FtsL was important for the interactions with FtsQ (Figure 4B). Given the predicted ΔΔG of ionic pairs, these are not the strongest interactions, but were relatively stable in the 10 ns of the dynamic simulation.

### Interface analysis

The FtsB/FtsL subcomplexes without the FtsQ molecule(s) have hydrogen bonds and salt bridges to stabilize the interface, but the main contribution to stability was always the hydrophobic contacts between leucine residues in the zipper motif. However, the number of stabilizing interactions was small for these long complexes, because the hydrophobic interactions came almost exclusively from the *a *position in the heptad. The position *d*, which theoretically must be hydrophobic, had several polar substitutions, resembling a multimeric coiled-coil motif [38-40]. In the simulated annealing step of the FtsB/FtsL models (without FtsQ), instability of these structures was found. The *rmsd *of the final structure was 6-10 Å (depending on the stoichiometry), with a weakening of *van der Waals *energies with respect to the initial structure. The polar interactions (hydrogen bonds and electrostatic) also seem to diminish during the course of the dynamic simulation (data not shown). This is in agreement with experimental data in *B. subtilis *proteins that show that the FtsB/FtsL interaction is not spontaneous when only the periplasmic/extracellular domains are used [34,44].

The interface analysis of the FtsB/FtsL/FtsQ models showed different results. For the unfavoured tetrameric models, presenting a three-helix coiled-coil fold between FtsB and FtsL (2:1:1 and 1:2:1 FtsB:FtsL:FtsQ), 20% of the interface residues and 40-50% of residues exposed to the solvent were hydrophobic (Tables 1 and 2). Additionally, there are few hydrogen bonds and salt bridges that stabilize the surface contact between the FtsB/FtsL subcomplex and FtsQ, especially in the 2FtsB:1FtsL:1FtsQ conformation, where the percentage of ASA (accesible surface area) in the interface was only 6%. These results confirm the conclusion derived from the molecular dynamic simulation showing that the tetrameric complexes that contain FtsQ and heterotrimeric FtsB/FtsL (2:1 or 1:2) could be less stable and less soluble due to the large number of surface hydrophobic residues and the small area of interaction, and would explain the null stability of the models in the molecular dynamic procedure [45,46].

**Table 2:** Surface distribution of amino acid residues

**FtsB:FtsL:FtsQ **^**a,b**^	**% Polar **^**c**^	**% Non polar **^**c**^	**% Charged **^**c**^
Trimeric (1:1:1)	30.63	38.38	31.00
Tetrameric (1:2:1)	34.10	42.30	23.60
Tetrameric (2:1:1)	32.50	45.80	21.70
Hexameric (2:2:2)	30.41	36.33	33.27
Mean stable complex ^d^	31.90	38.00	30.00

^a ^The interface parameters were obtained with the PROTORP server for the FtsB/FtsL/FtsQ complex after the molecular dynamic equilibration.

^b ^The interfaces considered were between the FtsB/FtsL subcomplex and FtsQ. The FtsB and FtsL pair was treated as a single unit.

^c ^The percentages of each kind of residue consider only the residues at the exposed surface.

^d ^The data for the mean stable complex were extracted from Reynolds et al. [65]

In the more stable trimeric and hexameric FtsB/FtsL/FtsQ models, the areas of the interface between FtsB/FtsL and FtsQ, were ~1.300 and ~5.000 Å^2^, respectively. In both models, a major proportion of polar and non-polar residues were found to be responsible for the hydrogen bonds and contacts between the proteins (Table 1 and 2). The number of these interactions and the proportion of non-polar, polar and charged residues at the interface of these models resemble that of a stable transient heteromultimer [45,47], suggesting that they could be close to the minimum energy structure.

Another important feature of the trimeric and hexameric FtsB/FtsL/FtsQ models is the proportion of non-polar residues at the interfaces, in both cases near 40%, a value found in many stable complexes. It has been shown that the strength of binding in transient dimers is related to the proportion of polar and non-polar residues at the interface of the proteins [45]. The "weak" interaction, in which the proteins exist as monomers or dimers at physiological concentrations, has a higher proportion of polar residues at the interface, and in general, a geometrically-planar surface of interaction is found. The "strong" interaction in a dimer involves a higher proportion of non-polar residues, and a more complex geometry of surface contact. The trimeric and hexameric models of FtsB/FtsL/FtsQ showed that the characteristic residue distribution in the surface between FtsB/FtsL and FtsQ is closer than that found in a strong transient interaction (Table 1, Table 2, Figure 4). It is tempting to speculate that the other polar residues and the hydrophobic residues exposed to the solvent could be useful for interaction with the other proteins of the *divisome *such as FtsW, FtsI or FtsN, because these percentages would be unusual for a very stable soluble multimeric complex [45]. Attending these criteria, the trimeric FtsB/FtsL/FtsQ model would be more plausible because of the higher percentage of non-polar residues in the exposed surface, compared to the hexameric model.

**Figure 6:**
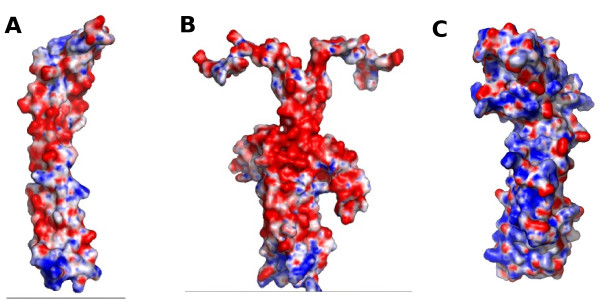
**Electrostatic potential surfaces of FtsB/FtsL subcomplexes and FtsQ**. Blue represents the positive charges while red indicates the negative charges. The map was calculated with an adaptive Poisson-Boltzmann solver software (APBS). A. 1FtsB:1FtsL model with two-helices in the coiled-coil fold. B. 2FtsB:2FtsL model with four-helices in the coiled-coil fold. C. FtsQ molecule in which the left side is the surface of the interaction with the FtsB/FtsL complex.

Besides the observed overall proportions of non-polar residues at the interfaces, there are zones in the complexes that showed a substantial presence of polar interactions rather than hydrophobic ones. The N-terminal zone that should be proximal to the membrane, is rich in salt bridges. This is mainly due to the amino acid contribution of FtsB and FtsL. Both proteins are rich in charged residues (30% and 26% respectively) with both positive and negative charges. The distribution of charges in the rest of the sequence is rather random in both proteins, but there is a zone in the middle section of the coiled-coil fold, between residues 50-70 of FtsB and 85-95 in FtsL where the predominant charge is negative. This "negative patch" in the structure of the complex is visible in the electrostatic surface of both trimeric and hexameric models, although it is more evident in the hexameric model (Figure 6). FtsQ also presented a high level of charged residues (24%), however it is more difficult to interpret this fact due to the more complex pattern of secondary and tertiary structure. The electrostatic potential surface showed clearly that one side of FtsQ is positively charged (Figure 6C), and that this side in both models faces and interacts with the FtsB/FtsL subcomplex, where the charge complementation helps to form the ternary complex. The overall level of conservation in the three proteins is low, but the relatively high presence of charged residues is a conserved feature, as is the "negative patch" in FtsB and FtsL (See Additional File 1: Figure S1 and S2). The electrostatic surface in the N-terminal of the modelled complexes tends to be slightly positive (Figure 6), complementing the negatively-charged surface of the outer leaflet of the *E. coli *inner membrane.

In many proteins complexes, there are contact points between pairs of residues that are crucial for the whole complex interactions, and that are identified by the change in binding energy in a mutation-by-alanine simulation. In both models of the FtsB/FtsL/FtsQ complex, specific and critical point interactions were absent. The search for "hot spots" in the interactions between the proteins by the alanine-mutation simulation resulted in a large number of contacts of discrete binding energy (Additional File 2: Table S1). This could be explained by the fact that FtsL and FtsB proteins have low residue similarity, preserving just the membrane topology, the genomic context and the leucine heptad [28]. The specificity of protein-protein interactions could be represented by the overall interface interaction rather than few contacts, and the surface topology of each protein could convert an unspecific interaction, such as a charge attraction (Figure 6), into a specific interaction. The binding strength could be reinforced by the transmembrane helices and the other proteins present in the *divisome*.

## Conclusions

The proposed FtsB/FtsL/FtsQ complex models are plausible because of the stability and the important number of hydrophobic residues accessible at the surfaces exposed to the solvent which could be available for the sequential interaction with the proteins of the peptidoglycan synthesis machinery. The very limited number of FtsQ molecules available for *divisome *formation in *E. coli *and the higher number of non-polar residues exposed to the solvent indicate that the trimeric FtsB/FtsL/FtsQ models are in accordance with the expected complex *in vivo*. The models proposed are likely not restricted to *E. coli *FtsQ, as important features are retained in homologues, even in the Gram-positive DivIB. Analysis with FtsQ of *Y. enterocolitica *indicates that the models are also applicable (data not shown). The proteins FtsB and FtsL also share their characteristics with their respective homologues, especially those related to the construction of the models. Numerous mutants have been described for FtsQ but only four have been used in this work as restrictions [15,22]. Others mutations like the related to septal localization (probably for the FtsK interaction) are those in residues Val92, Gln108, Val111 and Lys113 of the POTRA domain of FtsQ [15,19,22]. Coincidently in both models, these residues are in the exposed face of the POTRA domain on the opposite side from that which makes contact with the FtsB/FtsL subcomplex. Other mutations described to interrupt the FtsB or FtsL interaction, such as E176V or S166R [21] are on the opposite face of FtsQ with respect to the interaction with the FtsB/FtsL model. In other studies, the region of FtsQ which interacts with FtsB is between residues 136 to 202, and this is not compatible with our work where this region is on the opposite face [20]. Additional experimental analysis of the ternary complex structure could resolve the discordance between our results and previous findings. The interactions between FtsQ with FtsI were mapped in homologous proteins of *B. subtilis *(DivIB and PBP 2B) in the 229-257 region [48]. The corresponding region in *E. coli *FtsQ (129-155) is relatively free in both models and could establish the described interaction. Finally, as a projection of this work, the structure of the other proteins that interact in the periplasm could be used to construct a model of the complete complex of the peptidoglycan synthesis machinery. Furthermore, the detailed knowledge of the interactions between the division proteins could help to design new antibiotics targeted to disrupt specific protein-protein interactions, leading to novel and safe alternatives to known antibiotics.

## Methods

### Homology modelling

The structures of FtsB and FtsL proteins were built through structural homology using the software MODELLER 9.8 [49]. The template structures were coiled-coil proteins collected from threading servers: Pyre [50], 123D+ [51] and SAM-T08 [52]. The servers used to predict the secondary structure from multiple alignments were Jpred3 [53], SOMPA [54], PsiPRED [55] and HNN. In each modelling routine, 100 structures were generated without any restriction or special condition applied.

For the construction of FtsB dimers, two coiled-coil DNA-binding proteins were used [PDB:1T2K, PDB:1HKB]. For the trimeric models, several coiled-coil proteins were used [PDB:1AQ5, PDB:1M7L, PDB:1CE0]. For the tetramer, a very stable tetrameric form of mutant GCN4 was used [PDB:1GCL]. In all cases the alignment between the query and the template sequences was constructed manually, carefully locating leucine and other residues following the general rules observed in coiled-coil proteins [38,39].

To select the correct models, several methods were used. The statistical potentials DOPE software [56], included in the MODELLER suite, was used for a first classification. Then, an empiric residue pair potential matrix was used to evaluate the models. This matrix was constructed following the studies of Moont [57], but using 53 non-redundant structures from the coiled-coil family in the SCOP database. The reasoning behind this method was to apply a biological filter, reflecting the typical interaction in this kind of proteins. Through the combined use of these two scoring systems, ten models were chosen. These models were further analysed with Verify3D [58], PROCHECK [59] and PROSA II [60], selecting one model.

### Docking of FtsQ to the FtsB/FtsL complex

For protein-protein docking, the program 3D-Dock was used, which is based on the FTDock algorithm [61]. This software searches the right combination of positions through rigid-body surface complementation generating 10.000 possible models, and uses an empiric residue pair potential matrix to evaluate the models. Finally, two biological restrictions were used to select the final structure: the correct N-terminal-membrane position of all molecules and a distance restraint of 6 Å between the residues identified as interacting in FtsQ (Q232, D237, L259) and FtsB [15]. For FtsQ crystal available, the monomer structure was used, because the dimeric FtsQ structure observed was an artefact [15].

### Molecular dynamics

In all this work the GROMACS 4.0 software [62] was used. To perform MD simulation, the structure of the modelled complex was set to the GROMOS96 43a1 force field with explicit hydrogen atoms in the aromatic rings. The simulation cell was created in a triclinic periodic box with a minimum distance of 1.5 Å between the protein and the box walls. The complexes were solvated with approximately 40,000 water molecules, using the simple point charges (SPC) of the water model. The state of tritrable residues was defined with the H++ web server [63] and the necessary ionic species were added to neutralize the net charge. Electrostatic interactions were calculated using the Particle-Mesh Ewald method (PME) with a grid width of 1.2 Å and fourth-order spline interpolations. A cut-off distance of 9 Å was applied for Lennard-Jones interactions. The peptides and water were coupled together in a temperature bath with a v-rescale thermostat. When required, the pressure was coupled with an isotropic Berendsen barostat at a reference pressure of 1 bar. A 2 fs time step was used and an harmonic position restraint with a force constant of 1000 kJ mol^-1 ^nm^-2 ^in the z-axis and 500 kJ mol^-1 ^nm^-2 ^in the x and y-axis was applied to the heavy atoms of the N-terminal residues of all peptides. This position restraint was used for maintaining the correct membrane oriented proteins (described in the text). For equilibration of the complex in the aqueous media, the following procedure was performed: (1) 1.000 steps of steepest descent energy minimization *in vacuo*; (2) 1.000 steps of conjugated gradient energy minimization in water; (3) 1200 ps of simple simulated annealing from 200 K to 300 K; (4) 200 ps of NvT condition for correct adjustment of temperature; (5) 500 ps of NpT for density adjusting. The equilibrated structure obtained was subjected to 10 ns of NpT production dynamic at a coupled temperature of 310 K and 1 bar of pressure. All the bond lengths were constrained with the LINCS algorithm. The initial velocities of the atoms were taken from a Maxwell distribution at 298 K.

### Surface and Interface analysis

To analyse the properties and interactions at the interface of the distinct protein molecules of the complexes, two web servers were used. 1) the Alanine Scanning of Robetta server [64] was employed for the identification of "hot spots" in interfaces. It is based on a simple energy function developed primarily with empirical data extracted from crystallographic complexes deposited at PDB. 2) the PROTORP web-based server [65] was used. This determines the interface area by subtracting the accessible surface area (ASA) of the complex from that of the monomers, divided by two. Changes greater than 1.0 Å^2 ^indicate an interface residue. The hydrogen bonds were calculated with the program HBPLUS [66], and the salt bridges were calculated by selecting oppositely-charged atoms at least 4 Å apart [67]. The electrostatic potential surface was calculated with APBS and PDB2PQR software [68,69]. All the structures shown are displayed with PyMOL software (DeLano Scientific).

## Authors' contributions

FV performed the work. FV and OM analysed results and drafted the manuscript. AO helped to analyse results and correct the manuscript. JB, RL and OM conceived the study, and participated in its design and coordination. All authors read and approved the final manuscript and declare no conflict of interest.

## Supplementary Material

Additional File 1 **Sequence logo of the periplasmic region of FtsB and FtsL**. Figure S1: Sequence logo of the periplasmic region of FtsB. Around 100 sequences of FtsB and its Gram-positive bacteria equivalent, DivIC were aligned with Muscle software and the logo was obtained with HMMER software. The height of the symbols in the logo represents the level of conservation in the alignment. The upper symbol is the representative residue for that specific position. Figure S2: Sequence logo of the periplasmic region of FtsL. Around 100 sequences of FtsL were aligned with Muscle software and the logo was obtained with HMMER software. The height of the symbols in the logo represents the level of conservation in the alignment. The upper symbol is the representative residue for that specific position. Click here for file

Additional File 2 **Table S1: Selected Hot spot residues involved in binding contacts**. Hot spot residues were identified with the AlaScan server and the values of ΔΔG (kJ/mol) are reported. The type of interaction is shown: hydrogen bond (HB), salt bridge (SB) or van der Waals contact (VW) in the complex after the molecular dynamic equilibration. The letter in parenthesis (trimeric model) and the letter plus number in parenthesis (hexameric model) are the interacting partner chain identifier. Click here for file
