# Supplementary figures and images for: A model for the Escherichia coli FtsB/FtsL/FtsQ cell division complex
Source: BMC Struct Biol. 2011 Jun 14;11:28. doi: 10.1186/1472-6807-11-28 (PMC3152878; doi:10.1186/1472-6807-11-28)

Figure S1.

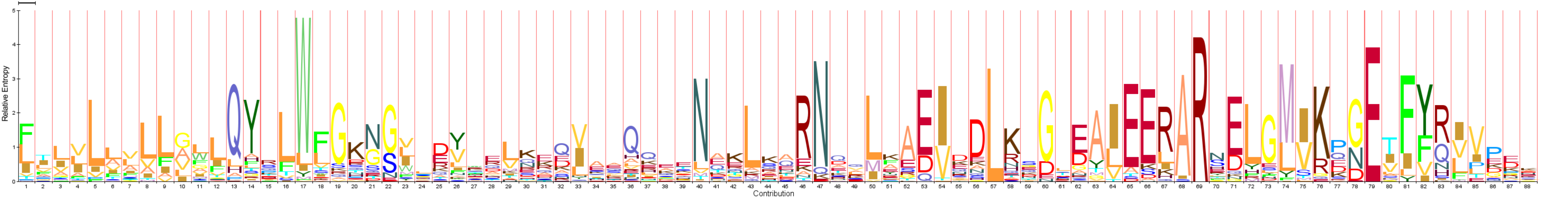

Figure S2.

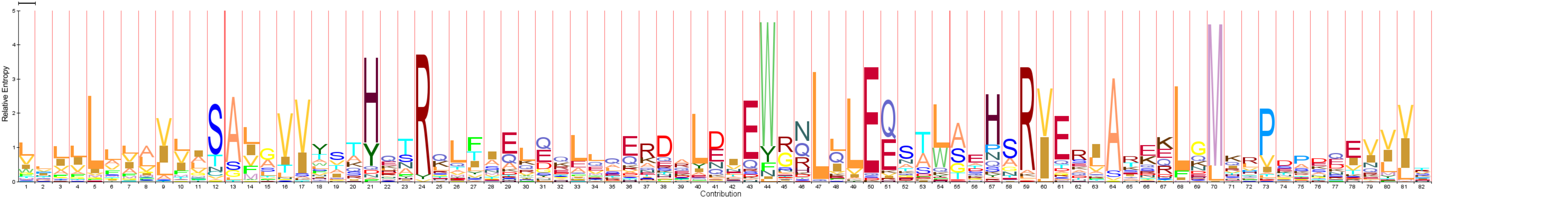

Supplement: Additional File 1 — Sequence logo of the periplasmic region of FtsB and FtsL. Figure S1: Sequence logo of the periplasmic region of FtsB. Around 100 sequences of FtsB and its Gram-positive bacteria equivalent, DivIC were aligned with Muscle software and the logo was obtained with HMMER software. The height of the symbols in the logo represents the level of conservation in the alignment. The upper symbol is the representative residue for that specific position. Figure S2: Sequence logo of the periplasmic region of FtsL. Around 100 sequences of FtsL were aligned with Muscle software and the logo was obtained with HMMER software. The height of the symbols in the logo represents the level of conservation in the alignment. The upper symbol is the representative residue for that specific position. [file 1472-6807-11-28-S1.PDF]
