# Supplementary material for: A model for the Escherichia coli FtsB/FtsL/FtsQ cell division complex
Source: BMC Struct Biol. 2011 Jun 14;11:28. doi: 10.1186/1472-6807-11-28 (PMC3152878; doi:10.1186/1472-6807-11-28)
Supplement: Additional File 2 — Table S1: Selected Hot spot residues involved in binding contacts. Hot spot residues were identified with the AlaScan server and the values of ΔΔG (kJ/mol) are reported. The type of interaction is shown: hydrogen bond (HB), salt bridge (SB) or van der Waals contact (VW) in the complex after the molecular dynamic equilibration. The letter in parenthesis (trimeric model) and the letter plus number in parenthesis (hexameric model) are the interacting partner chain identifier. [file 1472-6807-11-28-S2.PDF]

Table S1.

Trimeric model				Hexameric model			
**Residue**	**Chain**	**Interaction**	**ΔΔG**	**Residue**	**Chain**	**Interaction**	**ΔΔG**
Leu79	Q	VW(B)	1,57	Arg66	Q1	SB(B1)	2,70
Gln200	Q	HB(L)	3,68	Ile129	Q1	VW(L1)	1,80
Tyr243	Q	HB(L)	2,62	Thr236	Q1	HB(B2)	3,35
Leu259	Q	VW(B)	1,00	Asp35	B1	SB(Q1)	2,74
Asp150	Q	SB(B)	0,64	Glu65	B1	SB(Q2)	1,73
Glu134	Q	SB(B)	0,67	Arg72	B1	HB(Q2)	2,10
His27	B	SB(Q)	1,18	Glu74	B1	HB(Q2)	2,14
Arg31	B	SB(Q)	2,02	Thr83	B1	HB(Q2)	2,13
Met77	B	VW(Q)	1,04	Tyr85	B1	VW(Q2)	1,37
Arg86	B	HB(Q)	1,09	Arg82	L1	HB(Q1)	1,89
Leu87	B	VW(Q)	1,85	Arg96	L1	HB(Q1)	1,81
Asp34	B	HB(Q)	0,33	Val97	L1	VW(Q1)	2,14
Arg96	L	HB(Q)	1,26	Gln106	L1	HB(Q1)	2,21
Arg99	L	SB(Q)	1,22	Tyr68	Q2	VW(L2)	2,39
Ile100	L	VW(Q)	2,01	Arg175	Q2	SB(L2)	5,51
Glu103	L	SB(Q)	2,23	Arg185	Q2	SB(B1)	2,23
Gln108	L	HB(Q)	1,33	Gln232	Q2	HB(B1)	4,05
				Gln233	Q2	HB(B1)	2,89
				Trp256	Q2	VW(B1)	3,20
				Arg72	B2	HB(Q1)	2,71
				Tyr85	B2	VW(Q1)	2,90
				Ile85	L2	VW(Q2)	2,10
				Leu86	L2	VW(Q2)	3,04
				Asp93	L2	SB(Q2)	1,69
				Glu98	L2	SB(Q2)	4,14
				Leu105	L2	VW(Q2)	2,88
